# Viruses Without Borders: Global Analysis of the Population Structure, Haplotype Distribution, and Evolutionary Pattern of Iris Yellow Spot Orthotospovirus (Family *Tospoviridae*, Genus *Orthotospovirus*)

**DOI:** 10.3389/fmicb.2021.633710

**Published:** 2021-09-20

**Authors:** Afsha Tabassum, S. V. Ramesh, Ying Zhai, Romana Iftikhar, Cristian Olaya, Hanu R. Pappu

**Affiliations:** ^1^Department of Plant Pathology, Washington State University, Pullman, WA, United States; ^2^Indian Council of Agricultural Research-Central Plantation Crops Research Institute, Kasaragod, India

**Keywords:** BEAST, evolutionary genomics, gene flow, genetic differentiation, genetic recombination, iris yellow spot orthotospovirus, *in silico* RFLP, phylogenetics

## Abstract

Iris yellow spot, caused by Iris yellow spot orthotospovirus (IYSV) (Genus: *Orthotospovirus*, Family: *Tospoviridae*), is an important disease of *Allium* spp. The complete N gene sequences of 142 IYSV isolates of curated sequence data from GenBank were used to determine the genetic diversity and evolutionary pattern. *In silico* restriction fragment length polymorphism (RFLP) analysis, codon-based maximum likelihood studies, genetic differentiation and gene flow within the populations of IYSV genotypes were investigated. Bayesian phylogenetic analysis was carried out to estimate the evolutionary rate. *In silico* RFLP analysis of N gene sequences categorized IYSV isolates into two major genotypes *viz*., IYSV Netherlands (IYSV_*NL*_; 55.63%), IYSV Brazil (IYSV_*BR*_; 38.73%) and the rest fell in neither group [IYSV other (IYSV_*other*_; 5.63%)]. Phylogenetic tree largely corroborated the results of RFLP analysis and the IYSV genotypes clustered into IYSV_*NL*_ and IYSV_*BR*_ genotypes. Genetic diversity test revealed IYSV_*other*_ to be more diverse than IYSV_*NL*_ and IYSV_*BR*_. IYSV_*NL*_ and IYSV_*BR*_ genotypes are under purifying selection and population expansion, whereas IYSV_*other*_ showed decreasing population size and hence appear to be under balancing selection. IYSV_*BR*_ is least differentiated from IYSV_*other*_ compared to IYSV_*NL*_ genotype based on nucleotide diversity. Three putative recombinant events were found in the N gene of IYSV isolates based on RDP analysis, however, RAT substantiated two among them. The marginal likelihood mean substitution rate was 5.08 × 10^–5^ subs/site/year and 95% highest posterior density (HPD) substitution rate between 5.11 × 10^–5^ and 5.06 × 10^–5^. Findings suggest that IYSV continues to evolve using population expansion strategies. The substitution rates identified are similar to other plant RNA viruses.

## Introduction

Tospoviruses continue to be a major production constraint for a wide range of agronomic and horticultural crops worldwide (Gent et al., 2006; Pappu et al., 2009; Mandal et al., 2012; Mandal et al., 2012; Bag et al., 2015; Oliver and Whitfield, 2016; Turina et al., 2016; Resende et al., 2020). Iris yellow spot orthotospovirus (IYSV; genus: *Orthotospovirus*, family: *Tospoviridae*) (Resende et al., 2020) primarily infect *Allium* spp., which includes onion (*Allium cepa*), green onion (*Allium fistulosum)*, garlic (*Allium tuberosum*), leek (*Allium porrum*) (Gent et al., 2006; Cordoba-Selles et al., 2007; Bag et al., 2015; Karavina et al., 2016; Tabassum et al., 2016. The virus was first described in southern Brazil in 1981 on infected onion (*Allium cepa*; family: Amaryllidaceae) inflorescence stalks (scapes). The disease was referred to as “Sapeca.” In the US, the disease was first described in the Treasure Valley of southwestern Idaho and southeastern Oregon in 1989 (Gent et al., 2006). In 2003, the disease epidemic in Colorado (United States) caused a crop loss estimated at US $ 2.5–5 million (Gent et al., 2006). The disease has spread to most of the onion-growing areas in Africa, Asia, Europe, the Americas, and the Oceania (Centre for Agriculture and Bioscience International (CABI) - Invasive Species Compendium, 2019).

**FIGURE 1:**
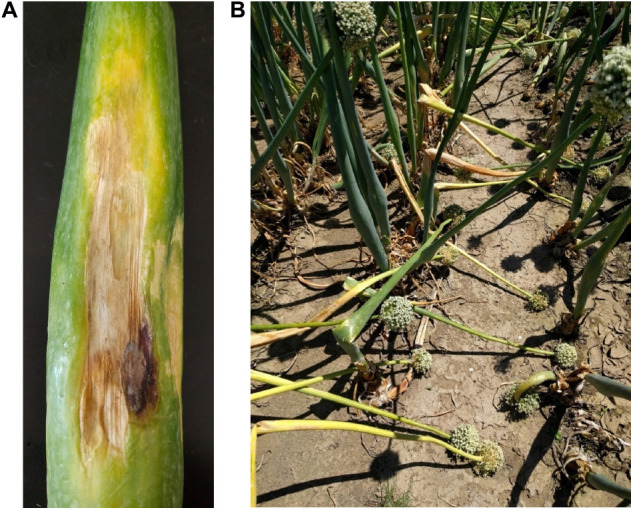
Symptoms associated with Iris yellow spot orthotospovirus infection on onion **(A)** on scapes; **(B)** toppling of the seed head.

The disease caused by IYSV is characterized by chlorotic or necrotic, straw-colored to white, dry, elongated or spindle shaped lesions along the scape (Figure 1A). Lesions are frequently at middle to lower portions of the scape. The diamond-shaped lesions tend to be less defined on leaves (Pappu et al., 2008; Bag et al., 2015). The photosynthetic activity is affected in the infected plants leading to reduced bulb size. As the disease progresses, the lesions girdle the scape causing the seed head to collapse leading to severe crop losses (Figure 1B; Gent et al., 2006).

IYSV, as other tospoviruses, consists of a tripartite genome: Small (S) and Medium (M) RNAs encode proteins in both sense and antisense orientations (ambisense) while the Large (L) RNA encodes protein from negative sense strand. The L RNA codes for RNA dependent RNA polymerase (RdRp), M RNA codes for glycoprotein precursors (G_*N*_ and G_*C*_) and the non-structural movement protein (NSm), and the S RNA codes for nucleocapsid (N) and non-structural silencing suppressor protein (NSs) (reviewed in Bag et al., 2015; Pappu et al., 2020; Resende et al., 2020).

Genetic evolution of viruses directly impacts the host-virus interactions and as such is important to ascertain genetic diversity within a viral species (Sacristan and Garcia-Arenal, 2008; Gibbs and Ohsima, 2010). Genetic drift, migration, mutation, natural selection, segment reassortment, and recombination are the major sources of evolutionary changes in the genetic architecture of viral populations (Moya et al., 2004; Butkovic et al., 2021). Phylo-geographical analysis is a powerful tool to determine the geographical distribution pattern of virus, assessing their genetic variation, and historical events that are shaping the genetic architecture of the viral populations (Hewitt, 2004; Chen et al., 2012). Comprehensive genetic architecture and evolutionary genomic analysis of viral populations have become a subject of increasing attention in a number of viruses.

While IYSV is widely distributed in the world, the complete genome of only a few isolates are sequenced. Since the N gene is considered as one of the descriptors for tospovirus identification and classification, the N gene of a large number of isolates was sequenced and the genetic diversity was determined (de Avila et al., 1993; Pappu et al., 2006; Nischwitz et al., 2007; Iftikhar et al., 2014; Bag et al., 2015). The number of N gene sequences of IYSV isolates reported since the last study (Iftikhar et al., 2014) has been on the rise. Building on the earlier findings, we carried out a more detailed and a global analysis of the extent of genetic recombination, genetic diversity, genetic differentiation, and gene flow among different genotypes of IYSV isolates reported from different parts of the world. Further, Bayesian model-based coalescent approaches were used to gain insights into the molecular evolutionary pattern of IYSV population.

## Materials and Methods

### Data Source of Nucleocapsid (N) Gene Sequences

Complete nucleocapsid (N) gene sequences of 142 IYSV isolates reported from across the globe were obtained the nucleotide sequence repository, GenBank. IYSV isolates analyzed were from 19 countries spread over six continents—Africa, Asia, Australia and New Zealand, Europe, North America (Canada, Mexico and the United States) and South America, infecting 10 different hosts including *Allium cepa* (the most commonly reported host), *Allium porrum, Eustoma russellianum, Allium tuberosum, Allium chinense*, Wild onion, *Alstroemeria* sp., *Allium sativum*, and *Allium fistulosum*. The N gene sequence (HQ267713) derived from tomato spotted wilt orthotospovirus (TSWV) infecting pepper crop in South Korea was used as an outgroup (Supplementary Table 1). Only complete IYSV N gene sequences (822 nt-long open reading frame coding for a 273-amino acid protein) were considered for analysis.

### *In silico* Restriction Fragment Length Polymorphism (RFLP) Analysis

N gene sequences were analyzed for sequence variations by performing *in silico* RFLP analysis using Restriction Mapper (Restriction Mapper, 2009). The complete N gene sequence was virtually digested, and sites were mapped as recognized by restriction enzyme *Hin*fI (Zen et al., 2005). Based on *Hin*fI digestion, IYSV isolates can be grouped into IYSV Netherlands (IYSV_*NL*_) or IYSV Brazil (IYSV_*BR*_) types. The size of the largest fragment generated by digestion is considered for differentiating the given isolate into two groups. The genotypes *viz.*, IYSV_*NL*_ and IYSV_*BR*_ are differentiated based on the resultant 308 and 468 bp fragments, respectively. Those isolates that yielded any different fragment size upon restriction digestion were grouped into IYSV_*other*_.

### Phylogeny Construction

Multiple sequence alignment (MSA) was performed using MUSCLE algorithm available in MEGA7 (Edgar, 2004). Best-fit model of nucleotide substitution was determined using MODEL TEST in MEGA7. Aligned sequence relatedness was evaluated using the Maximum Likelihood (default parameters with 1,000 bootstrap replicates) method based on Tamura parameter 3 model (T92) with Gamma distributed (G) available in MEGA7 (Kumar et al., 2016). The phylogenetic tree was rooted using TSWV N gene reported from South Korea as an outgroup.

### Population Selection Studies and Neutrality Test

Mean rates of non-synonymous (dN) and synonymous substitutions (dS) were calculated using codon-based maximum likelihood methods, i.e., SLAC (single like ancestor counting), FEL (fixed effects likelihood), and REL (random effects likelihood). DATAMONKEY server (Weaver et al., 2018) was used to calculate dN/dS ratio. To test the theory of neutral evolution, the test statistics such as Tajimas’s D (Tajima, 1989), Fu and Li’s D, and Fu and Li’s F (Fu and Li, 1993; Fu, 1997) were computed in DnaSP software.

### Genetic Differentiation and Gene Flow Estimates

DnaSP was used to compute nucleotide test statistics such as Ks, Kst (Kst value close to zero indicate no differentiation), Snn (Snn value close to one indicates differentiation) (Hudson, 2000) and haplotype statistics Hs, Hst (Hudson et al., 1992a,b). These tests estimate genetic differentiation within the populations of IYSV genotypes. Fst statistics was used to estimate the extent of the gene flow (panmixia or free gene flow has values close to zero whereas infrequent gene flow attains values close to one) (Hudson et al., 1992b).

### Recombination Detection Analysis (RDA)

Unaligned sequences were loaded in SDT v1.2 program, pairwise scan was performed with the MUSCLE, and the sequence data was saved with minimum identity of 70% and maximum of 100% to ensure sequences were properly aligned. The aligned IYSV N sequences were then used as an input query and analyzed for recombination events using Recombination Detection Program (RDP) v 4.0 (Martin and Rybicki, 2000), BOOTSCAN (Salminen et al., 1995), 3SEQ, GENECONV (Sawyer, 1999), MAXCHI (Maynard, 1992), CHIMAERA (Posada and Crandall, 2001) and SISCAN (Gibbs et al., 2000) available in RDP 4 Beta 4.88. Default settings for the different recombination detection methods and a Bonferroni corrected *P*-value cut-off of 0.05 were used for analysis.

### Recombination Analysis Tool (RAT)

Recombination analysis tool (RAT) was used for the analysis of aligned nucleotide sequences (Etherington et al., 2005). RAT algorithm uses pairwise comparisons between sequences based on the distance method to identify recombinants in nucleotide sequence alignment. Percentage of nucleotide similarities were compared using a sliding window size of 10% of the sequence length and an increment size being half of the window size.

### Bayesian Evolutionary Analysis by Sampling Trees (BEAST)

Bayesian phylogenetic analysis was performed in BEAST v2.4.6 (Bouckaert et al., 2014) to estimate evolutionary rate. Strict, relaxed (exponential, lognormal) and random local clocks were utilized for comparison (Bouckaert et al., 2014). Demographic models—coalescent constant population, coalescent exponential population, coalescent Bayesian skyline and coalescent extended Bayesian skyline were used to infer demographic history. “Temporal signal” (i.e., genetic changes between sampling times are sufficient and there is statistical relationship between genetic divergence and time) in the dataset was assessed using TempEst program (Rambaut et al., 2016). Using Markov Chain Monte Carlo (MCMC) method Bayesian phylogenies were constructed in BEAST v2.4.6. First 10% of the samples were discarded as burn-in. Convergence of the chain to stationary distribution and adequate sampling were analyzed using Tracer v1.6 (Tracer, 2018). Tracer was used to analyze the Effective Sample Size (ESS) and other prior parameter values. Tree Annotator was used for generating Maximum Clade Credibility (MCC) phylogenetic trees with common heights node. FigTree (2018) was used to generate the dendrograms.

## Results

### *In silico* Restriction Fragment Length Polymorphism Analysis

**FIGURE 2:**
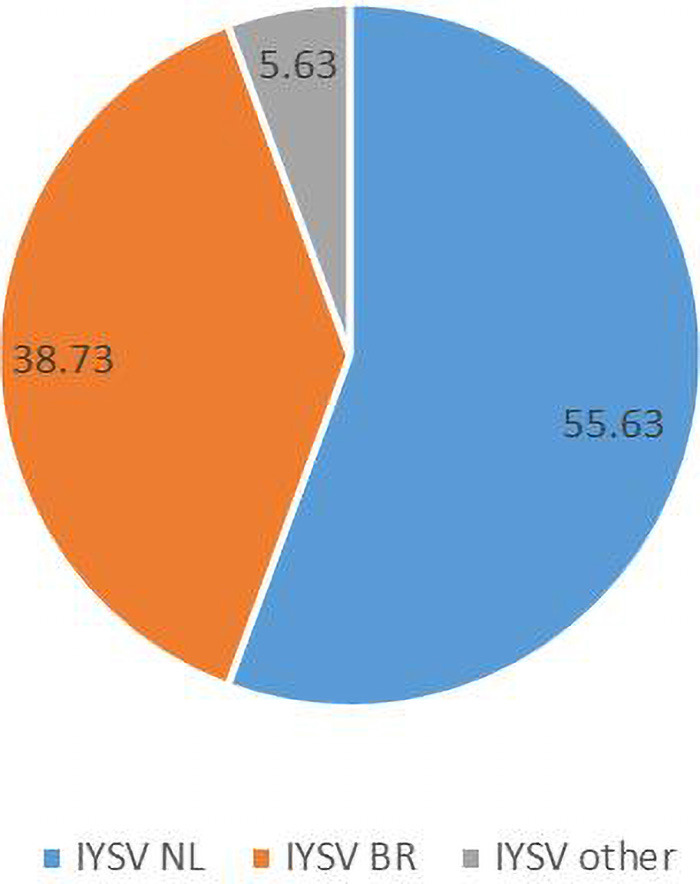
Relative distribution of the global collection of Iris yellow spot orthotospovirus genotypes (indicated as percentages) based on Restriction Fragment Length Polymorphism.

Computational RFLP-based analysis of N gene sequences recognizing *Hin*fI restriction site divided the population into two major groups [79 NL (55.63%), 55 BR (38.73%)]. Thus, the genotype IYSV_*NL*_ was found to be predominant over IYSV_*BR*_ while the rest (5.63%) fell in neither category (IYSV_*other*_) (Figure 2).

### Molecular Phylogeny of IYSV N Gene

**FIGURE 3:**
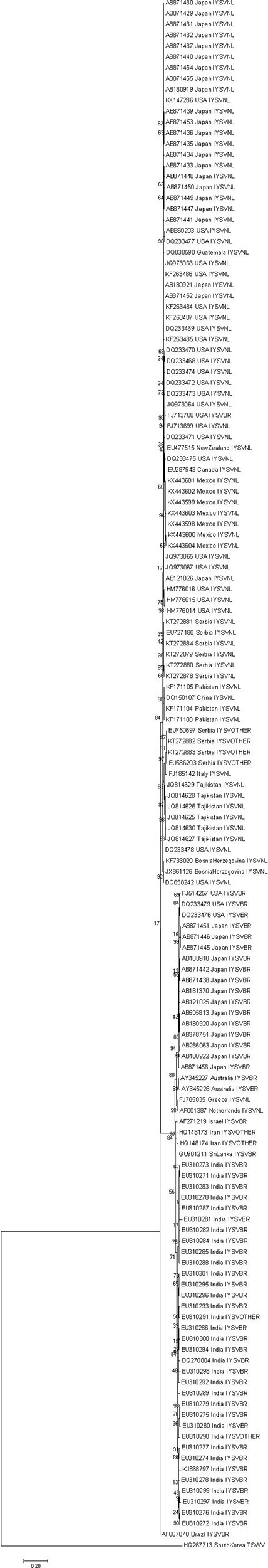
Dendrogram based on the nucleocapsid (N) gene nucleotide sequences of Iris yellow spot orthotospovirus (IYSV). Tomato spotted wilt orthotospovirus N gene isolate HQ267713.1 from South Korea was used as an outgroup. GenBank accession number, place of origin, and IYSV genotype are the indicators of each IYSV isolate.

Phylogenetic tree of the N gene of IYSV constructed based on the aligned nucleotide sequences (Figure 3) using Maximum Likelihood method broadly clustered IYSV genotypes into two major clades (NL and BR types). Four IYSV_*other*_ isolates (EU750697, KT27882, KT272883, and EU586203) and one IYSV_*BR*_ isolate (FJ713700) clustered with the NL group. Similarly, four more IYSV_*other*_ isolates (HQ148173, HQ148174, EU310290, and EU310291) and two IYSV_*NL*_ isolates (FJ785835 and AF001387) clustered with the BR group. One IYSV_*BR*_ isolate from Brazil (AF067070) formed a separate monophyletic clade along with a TSWV N gene isolate HQ267713 from South Korea (Outgroup). The clades also followed a geographical pattern as majority of IYSV_*NL*_ genotypes are from North America and IYSV_*BR*_ are from the Asian countries. Only one IYSV isolate has been reported from Brazil which formed a separate monophyletic clade even though *in silico* RFLP characterized it as an IYSV_*BR*_ type.

### Population Selection Studies, Neutrality Test, and Genetic Diversity Test

Gene codons that are in positive or negative selection pressure provide knowledge regarding the molecular evolution pattern of the N gene. The mean dN/dS (dN—rate of non-synonymous substitutions and dS—rate of synonymous substitutions) for N gene accessions belonging to IYSV_*NL*_ group were found to be 0.192 with no positively selected codon site. SLAC methodology identified 20 negatively selected codons in the N gene of IYSV_*NL*_ type. The data set when analyzed by FEL methodology revealed one positively selected codon site (codon no. 139) against 62 negatively selected codons. The dN-dS (mean difference between dN and dS) was –0.803 based on REL analysis denoting that the codon sites are under purifying selection acting against deleterious non-synonymous substitutions (Table 1).

**TABLE 1:** Codon substitution in the nucleocapsid gene of Iris yellow spot orthotospovirus genotypes.

**Genotype**	**Positively selected codon positions**	**No. of negatively selected codons**	**ω = dN/dS**	**dN-dS**
IYSV_NL_	139^b^	20^a^	0.192444	–0.803
		62^b^		
		73^c^		
IYSV_BR_	139^b^	27^a^	0.19119	–0.813
	30^c^	63^b^		
	40^c^	07^c^		
	109^c^			
	139^c^			
	210^c^			
	225^c^			
IYSV_other_	270^c^	3^a^	0.172079	–0.805
		38^b^		
IYSV_All_	109^b^	54^a^	0.205279	–
	139^ab^	90^b^		

*dN, the number of non-synonymous substitutions per non-synonymous site; dS, the number of synonymous substitutions per synonymous site ω—Ratio of dN/dS from SLAC (single like ancestor counting) methodology, dN-dS obtained from REL (random effects likelihood).*

*^*a*^Codons identified by SLAC at a cut-off p-value 0.1.*

*^*b*^Codons identified by FEL at a cut-off p-value 0.1.*

*^*c*^Codons identified by REL at a cut-off Bayes factor value 50.*

*IYSV_*All*_ = IYSV_*NL*_, IYSV_*BR*_ and IYSV_*other*_.*

For N gene accessions derived from IYSV_*BR*_, the mean dN/dS was found to be 0.191 with no positively selected codon site. SLAC methodology identified 27 negatively selected codons. The data set when analyzed by FEL revealed one positively selected codon site (codon no. 139) against 63 negatively selected codons. The dN-dS was –0.813 based on REL analysis suggesting that the codon sites are under purifying selection acting against deleterious non-synonymous substitutions. Six positively selected codon (codon nos. 30, 40, 109, 139, 210, and 225) and seven negatively selected codons were identified by REL analysis, respectively (Table 1).

For N gene accessions of IYSV_*other*_ group, the mean dN/dS was found to be 0.172 with no positively selected codon site. SLAC methodology identified three negatively selected codons. FEL methodology revealed no positively selected codon site against 38 negatively selected codons. The dN-dS was –0.805 based on REL analysis and it denotes codon sites are under purifying selection acting against deleterious non-synonymous substitutions. One positively selected codon (codon no. 270) and zero negatively selected codons were identified by REL analysis, respectively (Table 1).

Nucleotide diversity (π) of IYSV_*B*__*R*_ was about two folds higher than that for IYSV_*NL*_ (0.04513 and 0.01990; respectively, Table 2). However, the highest nucleotide diversity among the IYSV isolates was found in IYSV_*other*_ (0.08042) indicating IYSV_*other*_ is more genetically diverse than the IYSV_*NL*_ and IYSV_*BR*_ (Table 2). Number of polymorphic sites (S) was 136 from the N gene sequences of eight isolates of IYSV_*other*_, IYSV_*NL*_ showed 189 polymorphic sites among the N gene sequences obtained from the 79 isolates, whereas IYSV_*BR*_ showed 230 polymorphic sites obtained from the 55 isolates (Table 2). IYSV_*other*_ is more diverse than IYSV NL and BR based on number of polymorphic sites (S).

**TABLE 2:** Genetic diversity test of Iris yellow spot orthotospovirus genotypes.

**Genotype**	**N**	**S**	**π**	**Hd**
IYSV_NL_	79	189	0.01990	0.982
IYSV_BR_	55	230	0.04513	0.999
IYSV_Other_	08	136	0.08042	0.964
IYSV_All_	142	317	0.07220	0.994

*N, Number of isolates; S, Number of polymorphic (segregating) sites; Hd, haplotype diversity; π, nucleotide diversity within species; IYSV_*All*_ = IYSV_*NL*_, IYSV_*BR*_ and IYSV_*other*_.*

### Neutrality Test

The test of neutral evolution analyzed based on the total number of mutations and segregating sites, revealed statistically significant and non-significant negative values of test statistic Tajimas’s D for IYSV_*NL*_ and IYSV_*BR*_, respectively (Tables 3, 4). It indicates the operation of purifying selection and population expansion in major IYSV genotypes (NL and BR). Similarly, negative values of other test statistics such as Fu and Li’s D and Fu and Li’s F also corroborate the above findings with regard to IYSV_*BR*_ and IYSV_*NL*_ genotypes. However, positive values of all the test statistics such as Tajimas’s D, Fu and Li’s D, and Fu and Li’s F with respect to the genotype IYSV_*other*_ indicate the decrease in population size and act of balancing selection.

**TABLE 3:** Neutrality test of Iris yellow spot orthotospovirus genotypes based on total number of mutations.

**Genotypes**	**Tajimas’s D**	**Fu and Li’s D**	**Fu and Li’s F**
IYSV_NL_	−2.14425*	–1.43877	–2.07919
IYSV_BR_	–1.24392	–1.92209	–1.98807
IYSV_Other_	1.08486	0.71725	0.89745

Calculated using total number of mutations. *statistically significant at P < 0.01.

**TABLE 4:** Neutrality tests of Iris yellow spot orthotospovirus genotypes based on total number of segregating sites.

**Genotypes**	**Tajimas’s D**	**Fu and Li’s D**	**Fu and Li’s F**
IYSV NL	−1.96208*	–1.83392	–2.26501
IYSV BR	–0.93734	–2.14943	–2.01338
IYSV Other	1.42119	0.69566	0.96703

Calculated using total number of segregating sites. *statistically significant at P < 0.01.

### Genetic Differentiation and Gene Flow

Haplotype-based statistics (Hs and Hst) and nucleotide-based statistics (Ks, Kst, Snn) were estimated to evaluate genetic differentiation between the IYSV genotypes (Table 5). The statistically significant test values of Ks, Kst and Z reveals strong genetic differentiation among the IYSV genotypes studied. Snn value close to one indicates genetic differentiation even though insignificant test statistical values were obtained. IYSV_*BR*_ is more differentiated from IYSV_*other*_ (Kst value of 0.03724^∗^) compared to IYSV_*NL*_ genotypes (0.21377^∗^) based on the Kst values. Fst values show that the extent of gene flow between major genotypes, IYSV_*BR*_ and IYSV_*NL*_, is relatively high than the gene flow between individual BR and NL genotypes with IYSV_*o*__*ther*_. Among the major genotypes, IYSV_*NL*_ shows greater gene flow with IYSV_*other*_ than IYSV_*BR*_.

**TABLE 5:** Gene flow and genetic differentiation of Iris yellow spot orthotospovirus genotypes.

**Genotypes**	**H_s_**	**H_st_**	**χ ^2^**	***P*-value**	**K_t_**	**K_s_**	**K_st_**	**S_nn_**	**Z**	**F_st_**
IYSV_BR_ vs. IYSV_NL_	0.98869	0.00484	134	0.0768	58.49254	2.85914*	0.21377*	0.97761	*7.53452	0.72066
IYSV_BR_ vs. IYSV_other_	0.99516	0.00331	63	0.3368	44.90220	3.43202*	0.03724*	0.95238	6.42171*	0.26181
IYSV_NL_ vs. IYSV_other_	0.98056	0.00427	87	0.0427*	24.75033	2.56320*	0.06501*	0.98851	7.09306*	0.35394

*H_*s*_, H_*st*_—measure genetic differentiation based on haplotype statistics.*

*Ks, Kst, Snn, Z—measure genetic differentiation based on nucleotide statistics.*

Fst—measures extent of gene flow. *statistically significant at P < 0.05.

### Recombination Detection Analysis

**FIGURE 4:**
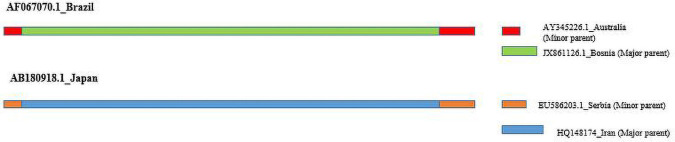
Potential recombinants detected in Iris yellow spot orthotospovirus nucleocapsid (N) gene by Recombination Detection Program (RDP4). Major and Minor parent for each recombinants are shown.

Three potential recombination events were detected among the IYSV N genes analyzed (Table 6 and Figure 4). AF067070 (BR type) IYSV isolate from Brazil is a potential recombinant of isolates: JX861126 Bosnia (major parent) and AY345226 Australia (minor parent). This recombinant was detected by GeneConv, 3Seq, algorithms in RDP. The recombination breakpoint begins at 789 in alignment (789 without gaps) with breakpoint clustering at 99% confidence interval ranging from 730 to 809 in alignment (730–809 without gaps) and breakpoint ends at 12 in alignment (12 without gaps) with breakpoint clustering at 99% confidence interval ranging from 822 to 44 in alignment (822–44 without gaps). The second recombination event involved isolate HQ148174 (IYSV_*other*_) from Iran putatively arising from EU310281 India (major parent) and AB180922 Japan (minor parent). However, only MaxChi algorithm detected this recombinant. The third recombinant, AB180918 (BR type) IYSV isolate from Japan, is the result of a potential recombination event 22 arising from HQ148174 Iran (major parent) and EU586203 Serbia (minor parent). This recombinant was detected by SiScan and 3Seq algorithms of recombination detection program. The recombination breakpoint begins at 20 in alignment (20 without gaps) with breakpoint clustering at 99% confidence interval ranging from 731 to 3 in alignment (731–3 without gaps) and breakpoint ends at 820 in alignment (820 without gaps) with breakpoint clustering at 99% confidence interval ranging from 731 to 3 in alignment (731–3 without gaps). Among the three putative recombinants detected, two belonged to IYSV_*BR*_ type and the remaining one belonged to IYSV_*other*_ type. IYSV isolate from Brazil is a potential recombinant and hence this isolate formed a separate monophyletic clade in the phylogenetic tree (Figure 3).

**TABLE 6:** Recombination events in Iris yellow spot orthotospovirus nucleocapsid (N) gene detected by Recombination Detection Program (RDP).

**Isolate**	**Parental isolate**		**Recombination Detection Program**	**Recombination event #**	***P*-values**
	**Major**	**Minor**			
AF067070_Brazil	JX861126_Bosnia	AY345226_Australia	GeneConv, 3Seq	1	3.193 × 10^–5^–1.333 × 10^–4^
HQ148174_Iran	EU310281_India	AB180922_Japan	MaxChi	2	–
AB180918_Japan	HQ148174_Iran	EU586203_Serbia	SiScan, 3Seq	22	2.879 × 10^–08^–9.951 × 10^–1^

### Recombination Analysis Tool

**FIGURE 5:**
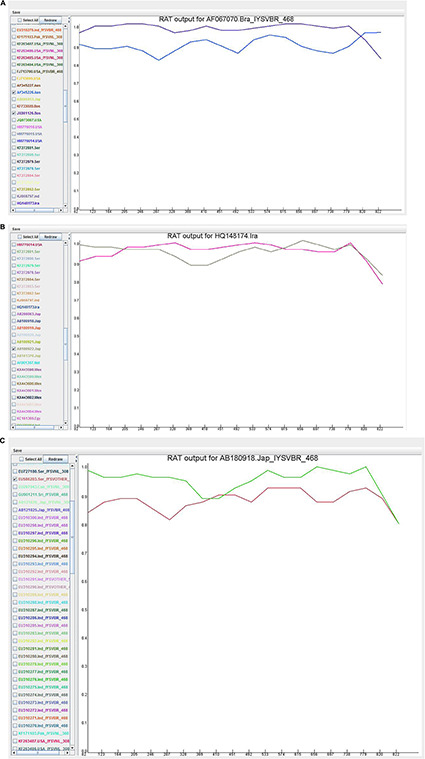
Recombination cross over sites in the Iris yellow spot orthotospovirus nucleocapsid (N) gene generated by Recombination Analysis Tool. The *X-*axis denotes sequence location along the genome and *Y*-axis represents the genetic distance. **(A)** AF067070_Brazil, the crossover site is located at 3′ end portion of the genome; **(B)** HQ148174_Iran, the crossover sites were located toward the 5′ and 3′ ends of the genome; **(C)** AB180918_Japan, the crossover site is located in the middle portion of the genome.

Recombination analysis tool (RAT) was used to substantiate the findings of the RDP. An isolate was considered recombinant when the major and minor parent isolates intersect at two points in the graph (Figure 5). Based on this criterion, HQ148174_Iran and AB180918_Japan were considered potential recombinants even though only MaxChi in the RDP4 program detected HQ148174_Iran as a recombinant.

### Bayesian Evolutionary Analysis by Sampling Trees (BEAST)

**FIGURE 6:**
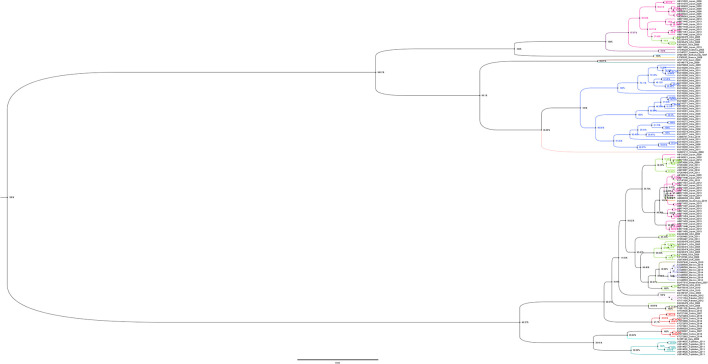
Maximum Clade Credibility (MCC) tree of Iris yellow spot orthotospovirus (IYSV) nucleocapsid (N) gene (nucleotide sequences) generated in BEAST v2.4.6. Nodes of IYSV isolates reported from different countries are colored.

The rates of nucleotide substitution in tospovirus genomes have not been reported. Therefore, the global repository of IYSV N gene sequences was used to estimate the rates of nucleotide substitution and discern the rapidity with which molecular evolution might occur in the tospoviruses. Genetic recombinants were removed for BEAST analysis since their inclusion violates the assumption of coalescent-based analyses and thus could result in incorrect estimates of the rate of evolution. For nucleotide models, Hasegawa-Kishino-Yano (HKY)-based analysis was performed and it converged satisfactorily. While comparing two models if the marginal posterior distributions of the log-likelihoods do not overlap then the model with the higher posterior distribution of log-likelihood was preferred. Estimate is a better approximation of the true posterior distribution when larger Effective Sample Size (ESS) is available (ESS > 200 are desirable). Based on the above criteria, General Time Reversible (GTR) relaxed exponential growth clock model with coalescent constant population was found to be the best fit with a marginal likelihood mean substitution rate of 5.08 × 10^–5^ subs/site/year, 95% highest posterior density (HPD) substitution rate between 5.11 × 10^–5^ and 5.06 × 10^–5^ and ESS was 305 (Supplementary Table 2). Bayesian phylogenetic tree separated the IYSV isolates into two distinct clades, clade I comprising of IYSV_*BR*_ isolates and clade II comprising IYSV_*NL*_ isolates. The isolates that belonged to the same geographic region (or same country) clustered together (Figure 6).

## Discussion

The global population structure and temporal dynamics of the IYSV conducted previously (Iftikhar et al., 2014) based on N gene sequences delineated that the viral isolates could be categorized into two major genotypes (IYSV_*BR*_ and IYSV_*NL*_). Further, temporal dynamics of IYSV showed greater incidence of IYSV_*BR*_ post-2005 compared to IYSV_*NL*_. Since the last publication, the number of N gene sequences added to the public repository has increased significantly. To gain a better understanding of the evolutionary genomics and to further gain deeper insights into the evolution rate of IYSV, we analyzed 142 complete N gene sequences using a wide range of computational tools to infer molecular evolutionary genomics.

*In silico* RFLP analysis to categorize the genotype of IYSV isolates showed that the majority of the isolates belonged to IYSV_*NL*_ category (55.63%), whereas 38.73% of IYSV_*BR*_ isolates were observed. There was an increment in IYSV_*NL*_ genotype incidence or characterization compared to IYSV_*BR*_ since the last report (Iftikhar et al., 2014). Interestingly, gene flow estimates showed greater gene flow between NL and BR genotypes, rather than between the individual major genotypes and the “other” category. Even between the major genotypes, IYSV_*NL*_ exhibited a greater gene flow with IYSV_*other*_. Also, a greater genetic diversity was observed in IYSV_*other*_, compared to NL and BR. However, codon substitution analysis of N gene showed little change since the last study (Iftikhar et al., 2014). In fact, the positively selected codons (codon positions 139 in BR and NL and 270 in IYSV_*other*_) remained intact despite the substantial increase in the number of isolates examined, suggesting the importance of these codon positions in improving the fitness of nucleocapsid protein. The negative selections in the other codon positions imply that the deleterious mutations in those positions are effectively removed in the IYSV population as a whole. Most of the codons of the N gene are neither under positive nor negative selection suggesting the neutral evolution of these codons.

Recombination is a common phenomenon in RNA viruses but the implications of recombination for evolution is not well studied (Sztuba-Solinska et al., 2011). There is a serious limitation in understanding the contribution of recombination to evolution of IYSV due to lack of full-length genome sequences. The potential recombinants identified in this study belonged to BR type which seems to be evolving using population expansion strategies. The recombination breakpoints were at 5’ and 3’ ends suggesting that these are potential hot spots for recombination (Gawande et al., 2015).

In the BEAST analysis, General Time Reversible (GTR) relaxed exponential growth clock model with coalescent constant population was found to be the best fit model explaining the genetic architecture of IYSV population. In a similar analysis of PVY genomic sequences, it was found that the relaxed uncorrelated log normal clock was the best fit with a population of constant size (Gibbs et al., 2017). Further, similar topology of the phylogenetic tree was obtained by both ML method and Bayesian MCC based phylogeny for IYSV.

PVY dating was reported by comparing the estimated phylogenetic dates with historical events in the worldwide adoption of potato and other PVY hosts (Gibbs et al., 2017). While the potato-PVY analysis was based on the sample collection dates over several decades, onion-IYSV interactions are relatively new and hence predicting the phylodynamic patterns and demographic history of IYSV require more such data on temporal scale. The PVY demographic history and population expansion was deduced and compared with that of geographic distribution of host (potato) suggesting direct influence of potato cultivation area on the population size of the virus (Mao et al., 2019). In this context, further studies how expansion of onion cultivation area influences the population expansion of IYSV will be interesting.

Bayesian coalescent estimates of evolutionary dynamics of citrus tristeza virus, based on the *p25* gene, showed that the rate of substitution was at 1.19 × 10^–3^ subs/site/year (Benítez-Galeano et al., 2017). Similarly, Bayesian phylogenetic reconstruction-based nucleotide substitution rates of CP gene derived from four species of viruses in *Secoviridae* family estimated it to range 9.29 × 10^–3^ to 2.74 × 10^–3^ (subs/site/year) (Thompson et al., 2014) while for tobamovirus the estimate ranged from 1 × 10^–5^ and 1.3 × 10^–3^ substitutions per site, per year (Pagan et al., 2010). Further, Bayesian analysis of VPg gene of PVY reveals that it has been evolving at a rate of 5.60 × 10^–4^ subs/site/year (Mao et al., 2019). Thus, the mean substitution rates identified for the IYSV N gene are comparable to those found in other plant-infecting RNA viruses. Substitution rates tend to be higher in RNA viruses as they are shown to mutate at faster rate. These mutations help in viral emergence on novel hosts but are not adaptive (Sacristan and Garcia-Arenal, 2008). Furthermore, the time of divergence of PVY clades, clade N and clade O, was found to be the year 1861 CE (95% credibility interval 1750–1948 CE) (Mao et al., 2019). Similar estimation of temporal divergence of IYSV_*BR*_ and IYSV_*NL*_ and the role of geographically driven adaptation of IYSV are worth exploring for a better understanding of the evolutionary dynamics of IYSV.

There are a very limited number of sequences of the other IYSV genes (NSm, NSs, G_*N*_/G_*C*_, RdRp) and even fewer complete genome sequences. Evolutionary analysis on such small sample size is not feasible. In the absence of complete genome sequences of a considerable number of the virus isolates (as is the case of IYSV) extrapolation of results of a single (or a few) gene(s) for the entire species is not uncommon. There are increasing number of studies on molecular evolutionary analysis, including phylodynamics and temporal evolutionary features of plant viruses based on a single or few viral gene sequences, such as VPg of PVY (Mao et al., 2019), NABP and CP genes of Potato virus M (PVM) (He et al., 2019) and P3, CI, Nib genes of (PVY) (Gao et al., 2020). However, to avoid any discrepancies in extrapolating the evolutionary analysis based on one or a few genes of a virus to the entire virus species, next generation sequencing–based sequencing followed by *de novo* assembly would provide a near complete genomic sequences that could be used to generate a more comprehensive picture of the genetic diversity of the virus populations (Zarghani et al., 2018).

## Conclusion

IYSV_*NL*_ was found to be the predominant genotype on a global scale. Interestingly, the IYSV_*other*_ genotype is genetically more diverse than IYSV_*BR*_ and IYSV_*NL*_ genotypes. Population structure analysis revealed that it is under purifying selection and the phenomenon of population expansion is occurring. BEAST-based molecular clock analysis showed that the rates of molecular evolution of IYSV N gene are similar to other plant RNA viruses. This study is a step forward in identifying molecular factors that contribute to the evolution of IYSV, and serves as a foundation for further evolutionary genomic studies on one of the economically important plant virus groups.

## Data Availability Statement

The raw data supporting the conclusions of this article will be made available by the authors, without undue reservation.

## Author Contributions

AT and HP conceived and designed the experiments. AT and YZ performed the experiments. AT, SR, YZ, and HP analyzed the data. AT, CO, SR, YZ, RI, and HP contributed reagents, materials, and analysis tools. AT, SR, and HP wrote the manuscript, proof-read and finalized the manuscript. All authors read and approved the final manuscript.

## Conflict of Interest

The authors declare that the research was conducted in the absence of any commercial or financial relationships that could be construed as a potential conflict of interest.

## Publisher’s Note

All claims expressed in this article are solely those of the authors and do not necessarily represent those of their affiliated organizations, or those of the publisher, the editors and the reviewers. Any product that may be evaluated in this article, or claim that may be made by its manufacturer, is not guaranteed or endorsed by the publisher.

## Abbreviations

IYSV: Iris yellow spot orthotospovirus
N gene: Nucleocapsid gene
RFLP: Restriction Fragment Length Polymorphism
RDP: Recombination Detection Program
RAT: Recombination Analysis Tool
BEAST: Bayesian Evolutionary Analysis by Sampling Trees.
