# Supplementary material for: Viruses Without Borders: Global Analysis of the Population Structure, Haplotype Distribution, and Evolutionary Pattern of Iris Yellow Spot Orthotospovirus (Family Tospoviridae, Genus Orthotospovirus)
Source: Front Microbiol. 2021 Sep 20;12:633710. doi: 10.3389/fmicb.2021.633710 (PMC8488366; doi:10.3389/fmicb.2021.633710)
Supplement: Supplementary file 1 [file Data_Sheet_1.docx]

**Supplementary Materials**

**Supplementary Table 1. Details of Iris yellow spot orthotospovirus Nucleocapsid (N) gene sequences used in the present study.**

| **S.No** | **GenBank accession** | **Host** | **Country** | **Year of collection (C)/ submitted to GenBank(S)/ released from GenBank(R)** |
| --- | --- | --- | --- | --- |
| 1 | AB378751.1 | *Allium cepa* | Japan | 2005C |
| 2 | JQ814630.1 | *Allium cepa* | Tajikistan | 2011C |
| 3 | JQ814629.1 | *Allium cepa* | Tajikistan | 2011C |
| 4 | JQ814628.1 | *Allium cepa* | Tajikistan | 2011C |
| 5 | JQ814627.1 | *Allium cepa* | Tajikistan | 2011C |
| 6 | JQ814626.1 | *Allium cepa* | Tajikistan | 2011C |
| 7 | JQ814625.1 | *Allium cepa* | Tajikistan | 2011C |
| 8 | FJ785835.1 | *Allium cepa* | Greece | 2008C |
| 9 | EU310301.1 | *Allium cepa* | India | 2008R |
| 10 | EU310299.1 | *Allium cepa* | India | 2008R |
| 11 | EU310284.1 | *Allium cepa* | India | 2008R |
| 12 | EU310279.1 | *Allium cepa* | India | 2008R |
| 13 | DQ838590.1 | *Allium cepa* | Guatemala | 2016R |
| 14 | JQ973066.1 | *Allium cepa* | USA | 2009C |
| 15 | JQ973065.1 | *Allium cepa* | USA | 2011C |
| 16 | JQ973064.1 | *Allium cepa* | USA | 2008C |
| 17 | EU750697.1 | *Allium cepa* | Serbia | 2007C |
| 18 | EU727180.1 | *Allium cepa* | Serbia | 2007C |
| 19 | EU586203.1 | *Allium cepa* | Serbia | 2007C |
| 20 | EU287943.1 | *Allium cepa* | Canada | 2010R |
| 21 | GU901211.1 | *Allium porrum* | Sri Lanka | 2009C |
| 22 | AB121026.1 | *Eustoma russellianum* | Japan | 2009R |
| 23 | AB121025.1 | *Eustoma russellianum* | Japan | 2009R |
| 24 | EU310300.1 | *Allium cepa* | India | 2011R |
| 25 | EU310298.1 | *Allium cepa* | India | 2011R |
| 26 | EU310297.1 | *Allium cepa* | India | 2011R |
| 27 | EU310296.1 | *Allium cepa* | India | 2011R |
| 28 | EU310295.1 | *Allium cepa* | India | 2011R |
| 29 | EU310294.1 | *Allium cepa* | India | 2011R |
| 30 | EU310293.1 | *Allium cepa* | India | 2011R |
| 31 | EU310292.1 | *Allium cepa* | India | 2011R |
| 32 | EU310291.1 | *Allium cepa* | India | 2011R |
| 33 | EU310290.1 | *Allium cepa* | India | 2011R |
| 34 | EU310289.1 | *Allium cepa* | India | 2011R |
| 35 | EU310288.1 | *Allium cepa* | India | 2011R |
| 36 | EU310287.1 | *Allium cepa* | India | 2011R |
| 37 | EU310286.1 | *Allium cepa* | India | 2011R |
| 38 | EU310285.1 | *Allium cepa* | India | 2011R |
| 39 | EU310283.1 | *Allium cepa* | India | 2011R |
| 40 | EU310282.1 | *Allium cepa* | India | 2011R |
| 41 | EU310281.1 | *Allium cepa* | India | 2011R |
| 42 | EU310280.1 | *Allium cepa* | India | 2011R |
| 43 | EU310278.1 | *Allium cepa* | India | 2011R |
| 44 | EU310277.1 | *Allium cepa* | India | 2011R |
| 45 | EU310276.1 | *Allium cepa* | India | 2011R |
| 46 | EU310275.1 | *Allium cepa* | India | 2011R |
| 47 | EU310274.1 | *Allium cepa* | India | 2011R |
| 48 | EU310273.1 | *Allium cepa* | India | 2011R |
| 49 | EU310272.1 | *Allium cepa* | India | 2011R |
| 50 | EU310271.1 | *Allium cepa* | India | 2011R |
| 51 | EU310270.1 | *Allium cepa* | India | 2011R |
| 52 | KF171103.1 | *Allium cepa* | Pakistan | 2012C |
| 53 | KF263487.1 | *Allium cepa* | USA | 2011C |
| 54 | KF263486.1 | *Allium cepa* | USA | 2011C |
| 55 | KF263485.1 | *Allium cepa* | USA | 2011C |
| 56 | KF263484.1 | *Allium cepa* | USA | 2011C |
| 57 | AF067070.1 | *Allium cepa* | Brazil | 1999R |
| 58 | FJ713700.1 | *Allium species* | USA | 2008C |
| 59 | FJ713699.1 | *Allium cepa* | USA | 2008C |
| 60 | FJ514257.1 | *Allium tuberosum* | USA | 2008C |
| 61 | AY345227.1 | *Allium cepa* | Australia | 2003R |
| 62 | AY345226.1 | *Allium cepa* | Australia | 2003R |
| 63 | AB505813.1 | *Allium chinense* | Japan | 2008C |
| 64 | KF733020.1 | *Allium cepa* | Bosnia and Herzegovina | 2012C |
| 65 | JX861126.1 | *Allium cepa* | Bosnia and Herzegovina | 2012C |
| 66 | JQ973067.1 | Wild Onion | USA | 2010C |
| 67 | HM776016.1 | *Allium cepa* | USA | 2010C |
| 68 | HM776015.1 | *Allium cepa* | USA | 2010C |
| 69 | HM776014.1 | *Allium cepa* | USA | 2010C |
| 70 | KT272881.1 | *Allium cepa* | Serbia | 2014C |
| 71 | KT272880.1 | *Allium cepa* | Serbia | 2014C |
| 72 | KT272879.1 | *Allium cepa* | Serbia | 2014C |
| 73 | KT272878.1 | *Allium cepa* | Serbia | 2014C |
| 74 | KT272884.1 | *Allium cepa* | Serbia | 2014C |
| 75 | KT272883.1 | *Allium cepa* | Serbia | 2014C |
| 76 | KT272882.1 | *Allium cepa* | Serbia | 2014C |
| 77 | KJ868797.1 | *Allium cepa* | India | 2014C |
| 78 | HQ148173.1 | *Allium cepa* | Iran | 2009C |
| 79 | HQ148174.1 | *Allium cepa* | Iran | 2009C |
| 80 | AB286063.1 | *Allium tuberosum* | Japan | 2006S |
| 81 | AB180918.1 | *Eustoma russellianum* | Japan | 2016R |
| 82 | AB180919.1 | *Allium cepa* | Japan | 2005R |
| 83 | AB180920.1 | *Allium cepa* | Japan | 2005R |
| 84 | AB180921.1 | *Eustoma russellianum* | Japan | 2005R |
| 85 | AB180922.1 | *Alstroemeria* | Japan | 2005R |
| 86 | AB181370.1 | *Alstroemeria* | Japan | 2008R |
| 87 | AF001387.1 | *Allium cepa* | Netherlands | 1997R |
| 88 | KX443598.1 | *Allium cepa* | Mexico | 2014C |
| 89 | KX443599.1 | *Allium cepa* | Mexico | 2014C |
| 90 | KX443600.1 | *Allium cepa* | Mexico | 2014C |
| 91 | KX443601.1 | *Allium cepa* | Mexico | 2014C |
| 92 | KX443602.1 | *Allium cepa* | Mexico | 2014C |
| 93 | KX443603.1 | *Allium cepa* | Mexico | 2014C |
| 94 | KX443604.1 | *Allium cepa* | Mexico | 2014C |
| 95 | DQ270004.1 | *Allium cepa* | India | 2005R |
| 96 | AF271219.1 | *Eustoma russellianum* | Israel | 2000S |
| 97 | FJ514257.1 | *Allium sativum* | USA | 2008C |
| 98 | DQ233479.1 | *Allium cepa* | USA | 2006R |
| 99 | DQ233476.1 | *Allium cepa* | USA | 2006R |
| 100 | DQ233478.1 | *Allium cepa* | USA | 2006R |
| 101 | DQ658242.1 | *Allium cepa* | USA | 2008R |
| 102 | KF171104.1 | *Allium cepa* | Pakistan | 2012C |
| 103 | KF171105.1 | *Allium cepa* | Pakistan | 2012C |
| 104 | DQ150107.1 | *Allium cepa* | Chile | 2005S |
| 105 | FJ185142.1 | *Allium cepa* | Italy | 2008C |
| 106 | ABB60203.1 | *Allium cepa* | USA | 2005S |
| 107 | DQ233469.1 | *Allium cepa* | USA | 2005S |
| 108 | EU477515.1 | *Allium cepa* | New Zealand | 2007C |
| 109 | DQ233476.1 | *Allium cepa* | USA | 2005S |
| 110 | DQ233471.1 | *Allium cepa var aggregatum* | USA | 2005S |
| 111 | DQ233470.1 | *Allium cepa* | USA | 2005S |
| 112 | DQ233474.1 | *Allium cepa* | USA | 2005S |
| 113 | DQ233473.1 | *Allium cepa* | USA | 2005S |
| 114 | DQ233472.1 | *Allium cepa* | USA | 2005S |
| 115 | AB871456.1 | *Allium cepa* | Japan | 2013S |
| 116 | AB871455.1 | *Allium cepa* | Japan | 2013S |
| 117 | AB871454.1 | *Allium cepa* | Japan | 2013S |
| 118 | AB871453.1 | *Allium cepa* | Japan | 2013S |
| 119 | AB871452.1 | *Allium cepa* | Japan | 2013S |
| 120 | AB871451.1 | *Allium cepa* | Japan | 2013S |
| 121 | AB871450.1 | *Allium cepa* | Japan | 2013S |
| 122 | AB871449.1 | *Allium cepa* | Japan | 2013S |
| 123 | AB871448.1 | *Allium cepa* | Japan | 2013S |
| 124 | AB871447.1 | *Allium cepa* | Japan | 2013S |
| 125 | AB871446.1 | *Allium cepa* | Japan | 2013S |
| 126 | AB871445.1 | *Allium cepa* | Japan | 2013S |
| 127 | AB871442.1 | *Allium cepa* | Japan | 2013S |
| 128 | AB871441.1 | *Allium cepa* | Japan | 2013S |
| 129 | AB871440.1 | *Allium cepa* | Japan | 2013S |
| 130 | AB871437.1 | *Allium cepa* | Japan | 2013S |
| 131 | AB871436.1 | *Allium cepa* | Japan | 2013S |
| 132 | AB871435.1 | *Allium cepa* | Japan | 2013S |
| 133 | AB871434.1 | *Allium cepa* | Japan | 2013S |
| 134 | AB871433.1 | *Allium cepa* | Japan | 2013S |
| 135 | AB871432.1 | *Allium cepa* | Japan | 2013S |
| 136 | AB871431.1 | *Allium cepa* | Japan | 2013S |
| 137 | AB871430.1 | *Allium cepa* | Japan | 2013S |
| 138 | AB871429.1 | *Allium cepa* | Japan | 2013S |
| 139 | AB871444.1 | *Allium cepa* | Japan | 2013S |
| 140 | AB871443.1 | *Allium cepa* | Japan | 2013S |
| 141 | AB871439.1 | *Allium cepa* | Japan | 2013S |
| 142 | AB871438.1 | *Allium cepa* | Japan | 2013S |
| 143 | HQ267713.1 | Pepper (TSWV) | South Korea | 2010C |

**Supplementary Table S2. Details of parameters estimated using BEASTv2.4.6 analysis**

|  | **Substitution model** | **Clock model** | **Population growth model** | **Sequence length (nt)** | **No. of sequences** | **Date range of sequences** | **Chain length (in millions)** | **Effective Sample Size** | **Marginal Likelihood Mean substitution rate** | **95% HPD^b^ substitution rate (subs/site/year)** |
| --- | --- | --- | --- | --- | --- | --- | --- | --- | --- | --- |
| 1. | GTR^a^ | Strict | Constant | 822 | 139 | 1997-2016 | 10 | 313 | 5.15x10^-5^ | 5.13x10^-5^ -5.17x10^-4^ |
| 2. | GTR | Strict | Exponential | 822 | 139 | 1997-2016 | 10 | 1172 | 5.15x10^-5^ | 5.17x10^-5^ -5.13x10^-5^ |
| 3. | GTR | Strict | Bayesian skyline | 822 | 139 | 1997-2016 | 10 | 569 | 5.15x10^-5^ | 5.17x10^-5^ -5.13x10^-5^ |
| 4. | GTR | Strict | Extended Bayesian skyline | 822 | 139 | 1997-2016 | 10 | 155 | 5.16x10^-5^ | 5.18x10^-5^ -5.14x10^-5^ |
| 5. | **GTR** | **Relaxed(exponential)** | **Constant** | **822** | **139** | **1997-2016** | **10** | **305** | **5.08x10^-5^** | **5.11x10^-5^ – 5.06x10^-5^** |
| 6. | GTR | Relaxed(exponential) | Exponential | 822 | 139 | 1997-2016 | 10 | 86 | 5.09x10^-5^ | 5.11x10^-5^ – 5.07x10^-5^ |
| 7. | GTR | Relaxed(exponential) | Bayesian skyline | 822 | 139 | 1997-2016 | 10 | 17 | 5.09x10^-5^ | 5.11x10^-5^-5.06x10^-5^ |
| 8. | GTR | Relaxed(exponential) | Extended Bayesian skyline | 822 | 139 | 1997-2016 | 10 | 382 | 5.08x10^-5^ | 5.11x10^-5^ – 5.06x10^-5^ |
| 9. | GTR | Relaxed(lognormal) | Constant | 822 | 139 | 1997-2016 | 10 | 239 | 5.10x10^-5^ | 5.12x10^-5^ – 5.07x10^-5^ |
| 10. | GTR | Relaxed(lognormal) | Exponential | 822 | 139 | 1997-2016 | 10 | 47 | 5.10x10^-5^ | 5.12x10^-3^ – 5.07x10^-5^ |
| 11. | GTR | Relaxed(lognormal) | Bayesian skyline | 822 | 139 | 1997-2016 | 10 | 86 | 5.10x10^-5^ | 5.13x10^-5^ – 5.08x10^-5^ |
| 12. | GTR | Relaxed(lognormal) | Extended Bayesian skyline | 822 | 139 | 1997-2016 | 10 | 236 | 5.10x10^-5^ | 5.13x10^-5^ – 5.08x10^-5^ |
| 13. | GTR | Random (local clock) | Constant | 822 | 139 | 1997-2016 | 10 | 44 | 5.13x10^-5^ | 5.15x10^-5^ – 5.11x10^-5^ |
| 14. | GTR | Random (local clock) | Exponential | 822 | 139 | 1997-2016 | 10 | 21 | 5.14x10^-5^ | 5.16x10^-5^ – 5.12x10^-5^ |
| 15. | GTR | Random (local clock) | Bayesian skyline | 822 | 139 | 1997-2016 | 10 | 42 | 5.14x10^-4^ | 5.16x10^-5^ – 5.12x10^-5^ |
| 16. | GTR | Random (local clock) | Extended Bayesian skyline | 822 | 139 | 1997-2016 | 10 | 8 | 5.15x10^-5^ | 5.18x10^-4^ – 5.12x10^-5^ |

^a^GTR – General Time Reversible model

^b^HPD – Highest Posterior Density

The best fit model is highlighted in bold text
